# A new analog of dihydroxybenzoic acid from *Saccharopolyspora* sp. KR21-0001

**DOI:** 10.3762/bjoc.20.44

**Published:** 2024-02-29

**Authors:** Rattiya Janthanom, Yuta Kikuchi, Hiroki Kanto, Tomoyasu Hirose, Arisu Tahara, Takahiro Ishii, Arinthip Thamchaipenet, Yuki Inahashi

**Affiliations:** 1 Department of Genetics, Faculty of Science, Kasetsart University, 50 Ngamwongwan Road, Ladyao, Chatuchak, Bangkok 10900, Thailand; 2 Ōmura Satoshi Memorial Institute, Kitasato University, 5-9-1 Shirokane, Minato-ku, Tokyo 108-8641, Japan; 3 Graduate School of Infection Control Sciences, Kitasato University, 5-9-1 Shirokane, Minato-ku, Tokyo 108-8641, Japan; 4 Faculty of Agriculture, University of the Ryukyus, 1 Senbaru, Nishihara, Okinawa 903-0213, Japan

**Keywords:** antioxidant activity, dihydroxybenzoic acid analog, rare actinomycetes

## Abstract

Actinomycetes are well-known as the main producers of bioactive compounds such as antibiotics, anticancers, and immunosuppressants. Screening of natural products from actinomycetes has been an essential part of several drug discovery programs. Finding such novel biologically active metabolites is immensely important because of their beneficial health effects. Recently, the discovery of new compounds has diverted attention to rare actinomycetes, since they are rich sources of natural products. In this study, a collection of rare actinomycetes at Kitasato University has been screened for potential novel compound producers. Among the rare actinomycetes, *Saccharopolyspora* sp. KR21-0001 isolated from soil on Ōha Island, Okinawa, Japan was selected as a potential producer. The strain was cultured in 20 L of production medium in a jar fermenter and the culture broth was extracted. Further purification revealed the presence of a new compound designated KR21-0001A (**1**). The structure was elucidated by NMR, and the absolute stereochemistry was determined by advanced Marfey’s method. The results indicated that **1** is a new analog of dihydroxybenzoic acid. **1** has no antimicrobial activity against bacteria and fungi but showed potent antioxidant activity_._

## Introduction

Actinomycetes are Gram-positive bacteria with high GC content in their genome. They are well-known as the main producers of bioactive compounds such as antibiotic, anticancer, antitumor, antiinflammatory, antihyperglycemic, antiapoptotic, immunosuppressant, and antioxidant compounds [1–3]. According to natural product screening since the 1920s, about 30,000 compounds derived from microbial sources have been reported [4]. Over 10,000 compounds are produced by actinomycetes, with 70% from *Streptomyces* and the rest from rare actinomycetes (non-*Streptomyces*) [5]. Rare actinomycetes are defined as actinomycete strains with low isolation rates when compared with the isolation of *Streptomyces* [6]. Currently, the discovery of new natural compounds is focusing on rare actinomycetes, which are believed to be rich sources of interestingly new compounds.

Bioactive compounds are vastly beneficial in medicine, pharmaceutical industry, and agriculture [7–8]. The thoughtful exploration of novel sources for acquiring new compounds is crucial, and the strategies employed in this pursuit are equally significant. One of the efficient approaches for finding new secondary metabolites from microorganisms is physicochemical (PC) screening. This approach involves the screening of the physicochemical properties of potential compounds, such as UV spectroscopy, mass spectrometry, and color reaction [9]. This technique has been used to find several new compounds from actinomycetes [10–13].

In the course of our PC screening for new natural products from rare actinomycetes, *Saccharopolyspora* sp. KR21-0001 produced a new compound **1**, whose physicochemical properties, accurate mass and UV spectrum, did not match any compound in the Dictionary of Natural Products database (version 32.1). Fermentation, isolation, structural elucidation, and biological activity of **1** are described in the following.

## Results and Discussion

**Figure 1:**
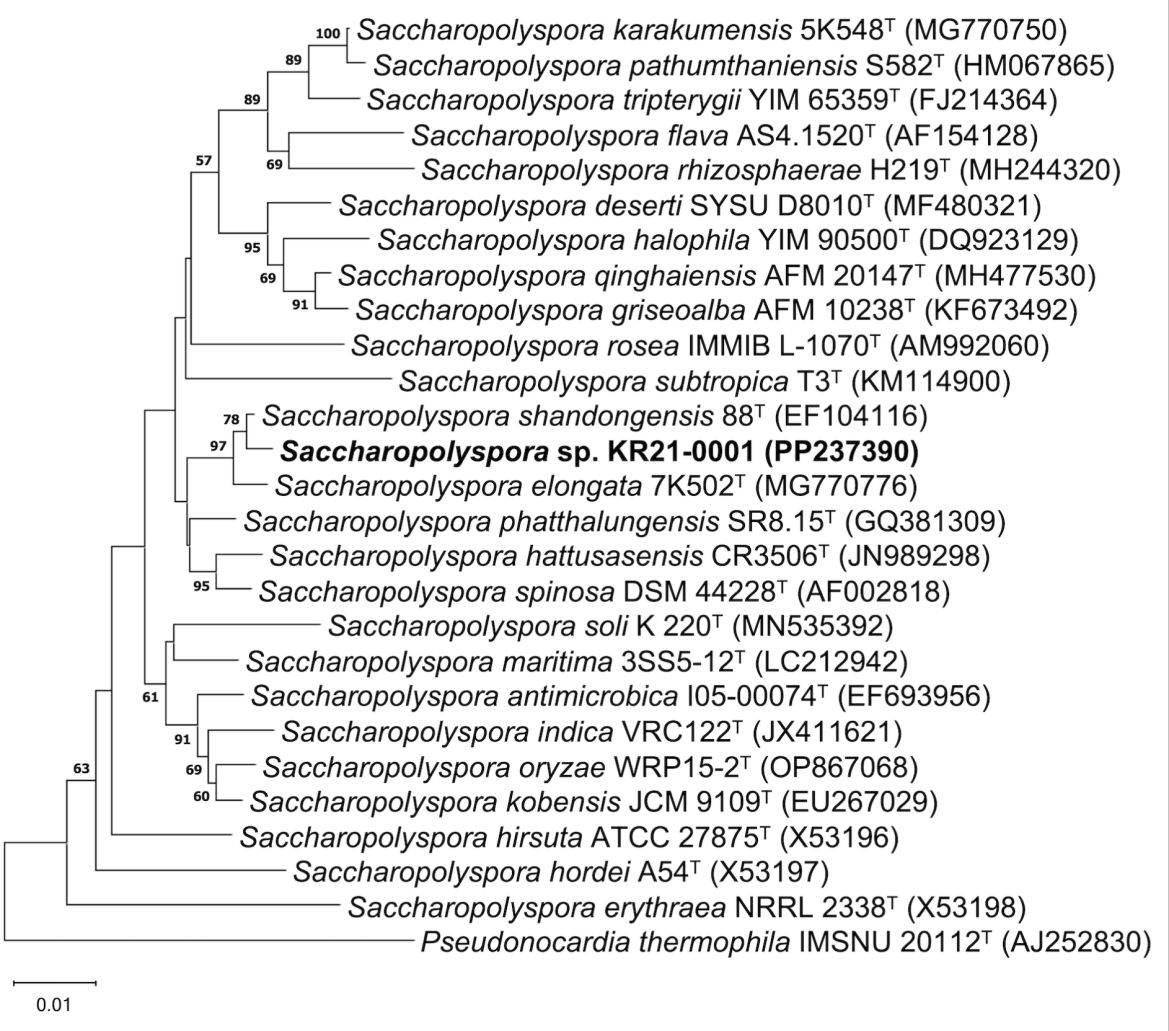
Neighbor-joining tree based on 16S rRNA gene sequences between KR21-0001 and members of the genus *Saccharopolyspora*. Only bootstrap values above 50% (percentages of 1000 replications) are shown.

*Saccharopolyspora* sp. KR21-0001 was isolated from soil in Ōha island, Okinawa, Japan. The 16S rRNA gene sequence (GenBank accession number: PP237390) showed the highest similarity values with *Saccharopolyspora shandongensis* 88^T^ (similarity: 99.6%) and *Saccharopolyspora elongate* 7K502^T^ (similarity: 99.0%). Phylogenetic analysis of the 16S rRNA gene sequences showed that the strain KR21-0001 was clustered with them (Figure 1). The similarity of 16S rRNA gene sequence and phylogenetic analysis indicated that strain KR21-0001 belongs to the genus *Saccharopolyspora*.

**Scheme 1:**
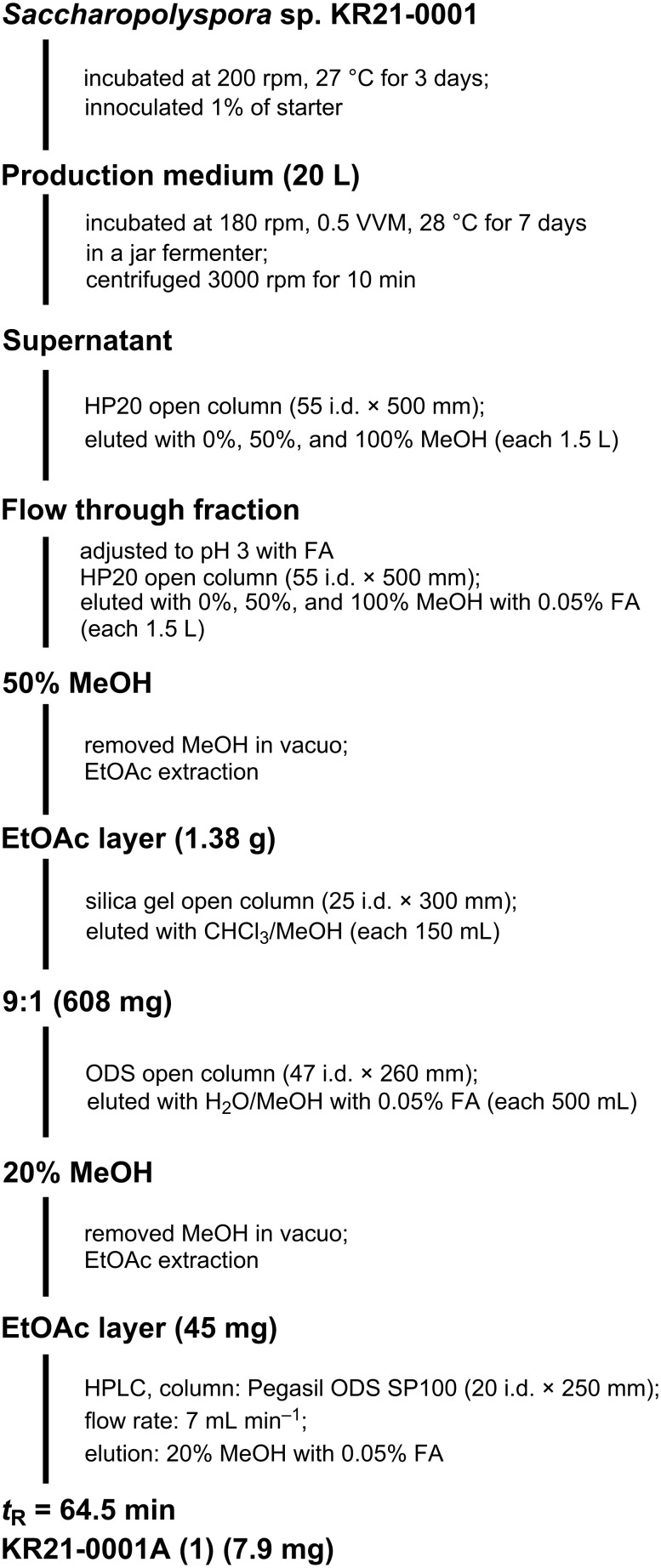
Fermentation of *Saccharopolyspora* sp. KR21-0001 and isolation procedure of KR21-0001A (**1**).

*Saccharopolyspora* sp. KR21-0001 was incubated in a jar fermenter containing 20 L of production medium for 7 days (Scheme 1). KR21-0001A (**1**) was purified from the culture broth guided by the ion peak (*m*/*z* 316) using LC–MS. The supernatant of the culture broth underwent HP20 column chromatography, but **1** was not retained in the resin and eluted in the flowthrough fraction. Since **1** was an acidic compound, the pH of the flowthrough fraction was adjusted to 3 with formic acid (FA) and chromatographed again on HP20 column. Now, **1** was retained in the resin and eluted by 50% MeOH with 0.05% FA. Then **1** was purified by silica gel and ODS column chromatography, and ethyl acetate extraction under acidic conditions. As the result of preparative HPLC of the crude extract, 7.9 mg of **1** was obtained (Scheme 1).

**Figure 2:**
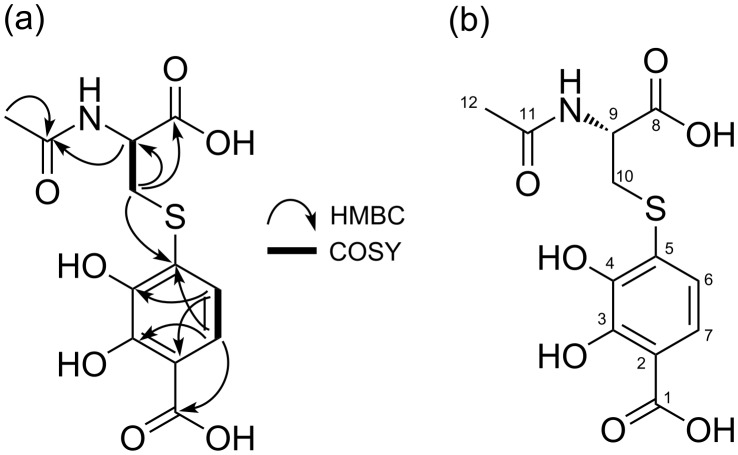
Structure of KR21-0001A (**1**). (a) ^1^H,^1^H COSY and HMBC correlations and (b) absolute configuration.

Table S1 (Supporting Information File 1) shows the physicochemical properties of **1**, which is a yellow oil soluble in MeOH and DMSO. The UV absorption maximum of **1** was at 286 nm (ε = 10238 M^−1^·cm^−1^). The molecular formula of **1** was determined as C_12_H_13_NO_7_S (requiring seven degrees of unsaturation) from the [M + H]^+^ ion at *m*/*z* 316.0484 (calcd. for C_12_H_14_NO_7_S, 316.0485) by HRESIMS. The ^1^H and ^13^C NMR spectral data of **1** are listed in Table 1. The ^1^H NMR and heteronuclear single quantum coherence (HSQC) data indicated the presence of two sp^2^ methines, one sp^3^ methine, one sp^3^ methylene, and one methyl group. The ^13^C NMR data showed the resonances of twelve carbons, which were classified into six olefinic carbons (including two oxygenated carbons: δ_C_ 151.0 and 145.2), three carbonyl carbons, one sp^3^ methine carbon, one sp^3^ methylene carbon, and one methyl carbon. A spin system H-6 (δ_H_ 6.85)/H-7 (δ_H_ 7.33) with an *ortho*-coupling constant (8.5 Hz) was observed using homonuclear correlation spectrometry (COSY). The heteronuclear multiple bond correlation (HMBC) cross-peaks from H-6 to C-2 (δ_C_ 112.4) and C-4 (δ_C_ 145.2), and from H-7 to C-1 (δ_C_ 173.8), C-3 (δ_C_ 151.0), and C-5 (δ_C_ 129.4) indicated that **1** has a 2,3-dihydroxybenzoic acid moiety (Table 1 and Figure 2a; Figure S3, Supporting Information File 1). A spin system H-9 (δ_H_ 4.58)/H_2_-10 (δ_H_ 3.54 and 3.23) was observed in ^1^H,^1^H COSY (Figure S5, Supporting Information File 1). The HMBC cross-peaks from H-9 to C-8 (δ_C_ 173.5) and C-11 (δ_C_ 173.4), from H_2_-10 to C-8 (δ_C_ 173.5) and C-9 (δ_C_ 53.6), and from H_3_-12 (δ_H_ 1.94) to C-11 (δ_C_ 173.4) indicated that **1** has a *N*-acetylcysteine moiety (Figure 2a). The HMBC cross-peak from H-10 (δ_H_ 3.23) to C-5 revealed the linkage between C-5 (δ_C_ 129.4) and C-10 through a sulfur atom. Based on these results, the structure of **1** was elucidated as 4-((2-acetamido-2-carboxyethyl)thio)-2,3-dihydroxybenzoic acid.

**Table 1:** NMR spectroscopic data of KR21-0001A (**1**) in CD_3_OD.

position	δ_C_, type	δ_H_ (*J* in Hz)	HMBC

1	173.8, C		
2	112.4, C		
3	151.0, C		
4	145.2, C		
5	129.4, C		
6	120.3, CH	6.85 d (8.5)	145.2, 112.4
7	121.5, CH	7.33 d (8.5)	173.8, 151.0, 129.4
8	173.5, C		
9	53.6, CH	4.58 dd (8.4, 4.4)	173.4, 173.5
10	34.2, CH_2_	3.54 dd (13.9, 4.4) 3.23 dd (13.9, 8.4)	173.4, 129.4 173.4, 129.4, 53.2
11	173.4, C		
12	22.3, CH_3_	1.94 s	173.4

The absolute configuration of **1** was determined by advanced Marfey’s analysis. **1** was de-sulfurized with skeletal Ni and then hydrolyzed with HCl. The obtained amino acid (alanine) and authentic amino acids were modified using *N*^α^-(5-fluoro-2,4-dinitrophenyl)-ᴅ-leucinamide (ᴅ-FDLA). The LC–MS analysis of ᴅ-FDLA derivatives revealed that the chiral center of the amino acid in **1** had ʟ configuration (Figure S7, Supporting Information File 1).

Antioxidant activity of **1** was measured via the DPPH radical. **1** showed potent DPPH radical scavenging activity with an IC_50_ value of 5.0 μg·mL^−1^, which is lower than that of trolox (IC_50_; 7.5 μg·mL^−1^) (Table 2). In contrast, **1** did not show antimicrobial activity against Gram-positive and Gram-negative bacteria and fungi at 20 µg per disc in the paper disc diffusion method.

**Table 2:** IC_50_ value of DPPH radical scavenging activity.

Sample	IC_50_ (µg·mL^−1^)

KR21-0001A	5.0
Trolox	7.5

Dihydroxybenzoic acid (DHBA) plays a role in anti-inflammatory, antihyperglycemic, antiapoptotic, and antioxidant processes [14]. 2,3-DHBA is found in nature and is produced by various plants (e.g., *Gentiana rigerscense* and *Vinca minor*), fungi (e.g., *Aspergillus sojae* and *Penicillium roquefortii*), and bacteria (e.g., *Acinetobacter calcoaceticus, Brucella abortus,* and *Bacillus* sp.) [15]. It is also known to be a component of some natural products, such as enterobactin, showing strong radical scavenging activity or antioxidant activity [16]. KR21-0001A (**1**) is a new analog of 2,3-DHBA connected to *N*-acetylcysteine (Figure 2b). **1** has a stronger antioxidant activity than trolox, which is a water-soluble analog of the free radical scavenger α-tocopherol [17–18]. **1** shows no antimicrobial activity against bacteria and fungi.

## Conclusion

Rare actinomycetes are excellent sources of novel bioactive compounds, since they are less explored for secondary metabolites than the more common strains of *Streptomyces* [19–20]. The compounds from this group often have unique structures that may exhibit novel biological activities and could be applied in various industries, such as pharmaceutics, agriculture, and environmental remediation [21–22]. However, the discovery of bioactive compounds from rare actinomycetes comes with challenges, such as the difficulty of isolation from environments. In this study, we searched for new secondary metabolites from the rare actinomycete *Saccharopolyspora* sp. KR21-0001. As a result, KR21-0001A (**1**), a new analog of dihydroxybenzoic acid, was discovered as a potent antioxidant. In conclusion, exploring rare actinomycetes for bioactive compound discovery represents a promising way in the quest for new drugs and biotechnological innovations. Continued research in this area has the potential to uncover valuable compounds that contribute to human health, pharmaceutical industry, and environmental sustainability.

## Experimental

### Taxonomic analysis

*Saccharopolyspora* sp. KR21-0001 was isolated from soil in Ōha Island, Ou, Kumejima, Shimajiri District, Okinawa, Japan. Genomic DNA was prepared, and the 16S rRNA gene was amplified by PCR using the method of Inahashi and co-workers [23]. The sequencing analysis was performed by Eurofins Genomics. Similarity of 16S rRNA gene sequence was computed by using EzBioCloud [24]. A phylogenetic tree was constructed based on the neighbor-joining method [25] by using MEGAX [26].

### Fermentation

*Saccharopolyspora* sp. KR21-0001 was inoculated into 100 mL of seed medium (2.4% soluble starch, 0.1% glucose, 0.3% peptone, 0.3% meat extract, 0.3% yeast extract, and 0.4% CaCO_3_; pH 7.0) as the starter and incubated at 180 rpm in a rotary shaker at 27 °C for 4 days. The 1% portion of the starter was transferred into 20 L of production medium (2% glycerol, 2% soybean meal, and 0.3% NaCl) in a jar fermenter and further incubated at 150 rpm, 27 °C, 0.5 VVM (volume per volume per minute) for 7 days.

### Purification and extraction

The 7-day culture broth of *Saccharopolyspora* sp. KR21-0001 was centrifuged at 3000 rpm for 10 min. The supernatant fraction was eluted stepwise with 1.5 L of MeOH/H_2_O (0%, 50%, and 100%) through a HP20 column (55 i.d. × 500 mm), and all fractions were analyzed by LC–MS. The flow-through and 0% fractions were mixed and adjusted to pH 3 by adding formic acid (FA). Then, the mixture was purified by eluting with MeOH/H_2_O (0%, 50%, and 100%) with 0.05% of FA through a HP20 column (55 i.d. × 500 mm). The 50% fraction was concentrated in vacuo to remove MeOH and extracted with ethyl acetate. The ethyl acetate layer was purified using a silica gel column (25 i.d. × 300 mm) by eluting with CHCl_3_/MeOH (100:0, 100:1, 100:2, 10:1, 1:1, and 0:100). The 10:1 fraction was purified with an ODS column (47 i.d. × 260 mm) by eluting with MeOH/H_2_O (0%, 10%, 20%, 30%, 40%, 50%, 60%, 80%, and 100%) with 0.05% FA. The 20% fraction was purified by HPLC (Shimadzu, Columbia, MD, U.S.A.) with an ODS column (Pegasil ODS SP100, 20 i.d. × 250 mm) at a flow rate of 7 mL·min^−1^ and eluted with 20% MeOH with 0.05% FA (Scheme 1). The fraction with a retention time of 64.5 min was concentrated in vacuo to yield **1** (7.9 mg).

### LC–MS analysis

Electrospray ionization-mass spectrometry (ESIMS) data was collected using a Triple TOF 5600+ LC–MS/MS System (AB Sciex, Framingham, MA, USA) with an ODS column (Capcell Core C18, 3.0 i.d. × 100 mm, 40 °C) (Osaka Soda, Japan) at a flow rate of 0.5 mL·min^−1^ and gradient elution with MeOH/H_2_O with 0.1% FA.

### Structure elucidation

Spectra from ^1^H NMR at 500 MHz and ^13^C NMR at 125 MHz were measured in CD_3_OD using a JNM-ECA500 (JEOL Ltd., Tokyo Japan). Chemical shifts were referenced to CD_3_OD (3.31 ppm) in the ^1^H NMR spectra and CD_3_OD (49.0 ppm) in the ^13^C NMR spectra. Infrared (IR) spectroscopy was carried out using an FT-4600 Fourier transform infrared spectrometer (JASCO P-2200 Polarimeter, Japan). The UV spectra were measured using a SpectraMax QuickDrop micro-volume spectrophotometer (Molecular Devices, LLC., San Jose, USA) at wavelengths of 200 to 800 nm. Optical rotation was measured using a P-2200 polarimeter (JASCO).

### De-sulfurization and hydrolyzation

KR21-0001A (**1**) (1 mg) was dissolved in EtOH (0.5 mL) and treated with skeletal Ni (1 mg). The reaction mixture was stirred for 13 h at room temperature under H_2_ (Figure S6, Supporting Information File 1). Then, the mixture was filtered through celite pad, and the filtrate was concentrated in vacuo. A volume of 100 µL of 1 M HCl was added and heated at 100 ºC for 3 h. After removing HCl in vacuo, the hydrolysate of de-sulfurized derivative of **1** was dissolved in 1 mL of H_2_O.

### Advanced Marfey’s analysis

A volume of 20 µL of 1 M NaHCO_3_ was added to each 50 µL of the hydrolysate of the de-sulfurized derivative of **1** and 1 mM standard amino acids (ʟ- and ᴅ-alanine). Then, 50 µL of ᴅ-FDLA (10 mg·mL^−1^ in acetone) was added to the mixtures and mixed well (the color changed from yellow to red). Next, the mixtures were incubated at 37 °C for 1 h. After incubation, 25 µL of 1 M HCl was added to each sample for neutralization. The mixtures were concentrated to dryness in vacuo, dissolved in acetonitrile, and analyzed by LC–MS.

### Biological activity

#### Antioxidant activity

The antioxidant activity of KR21-0001A (**1**) was measured using 2,2-diphenyl-1-picrylhydrazyl (DPPH) (Antioxidant Assay Kit, DOJINDO^©^ laboratory, Japan) according to the manufacturer’s protocol.

#### Antimicrobial activity

KR21-0001A (**1**) was investigated for antibacterial and antifungal activities with two strains of Gram-positive bacteria, *Kocuria rhizophila* ATCC 9341 and *Bacillus subtilis* ATCC 6633; two strains of Gram-negative bacteria, *Escherichia coli* Europe NIHJ and *Xanthomona campestis* pv. *oryzae* KB 88; and two strains of fungi, *Candida albicans* ATCC 64548 and *Mucor racemosus* IFO 4581. All strains were tested by paper disc diffusion assay at 20 µg on each paper disc and incubated at 37 °C for 24 h for *M. luteus*, *B. subtilis*, and *E. coli*; and 27 °C for 24 h for *X. oryzae*, *C. albicans*, and *M. racemosus*.

## Supporting Information

File 1 Additional data and NMR spectra.

## Data Availability

All data that supports the findings of this study is available in the published article and/or the supporting information to this article.
