# Supplementary material for: A new analog of dihydroxybenzoic acid from Saccharopolyspora sp. KR21-0001
Source: Beilstein J Org Chem. 2024 Feb 29;20:497–503. doi: 10.3762/bjoc.20.44 (PMC10910382; doi:10.3762/bjoc.20.44)
Supplement: File 1 — Additional data and NMR spectra. [file Beilstein_J_Org_Chem-20-497-s001.pdf]

BEILSTEIN JOURNAL OF ORGANIC CHEMISTRY

Supporting Information

for

A new analog of dihydroxybenzoic acid from *Saccharopolyspora* sp. KR21-0001

Rattiya Janthanom, Yuta Kikuchi, Hiroki Kanto, Tomoyasu Hirose, Arisu Tahara, Takahiro Ishii, Arinthip Thamchaipenet and Yuki Inahashi

Beilstein J. Org. Chem. **2024,** *20,* 497–503. doi:10.3762/bjoc.20.44

Additional data and NMR spectra

License and Terms: This is a supporting information file under the terms of the Creative Commons Attribution License (https://creativecommons.org/licenses/by/4.0). Please note that the reuse, redistribution and reproduction in particular requires that the author(s) and source are credited and that individual graphics may be subject to special legal provisions.
The license is subject to the *Beilstein Journal of Organic Chemistry* terms and conditions: (https://www.beilstein-journals.org/bjoc/terms)

Table contents

Figure S1: ^{1}H NMR spectrum of KR21-0001A (**1**) in CD_3OD

Figure S2: ^{13}C NMR spectrum of KR21-0001A (**1**) in CD_3OD

Figure S3: HMBC spectrum of KR21-0001A (**1**) in CD_3OD

Figure S4: HSQC spectrum of KR21-0001A (**1**) in CD_3OD

Figure S5: $^{1}H,^{1}H$ COSY spectrum of KR21-0001A (**1**) in CD_3OD

Figure S6: De-sulfurization and hydrolyzation of KR21-0001A (**1**)

Figure S7: LC/MS analyses of the D-FDLA-derivatives

Table S1: Physicochemical properties of KR21-0001A (**1**)

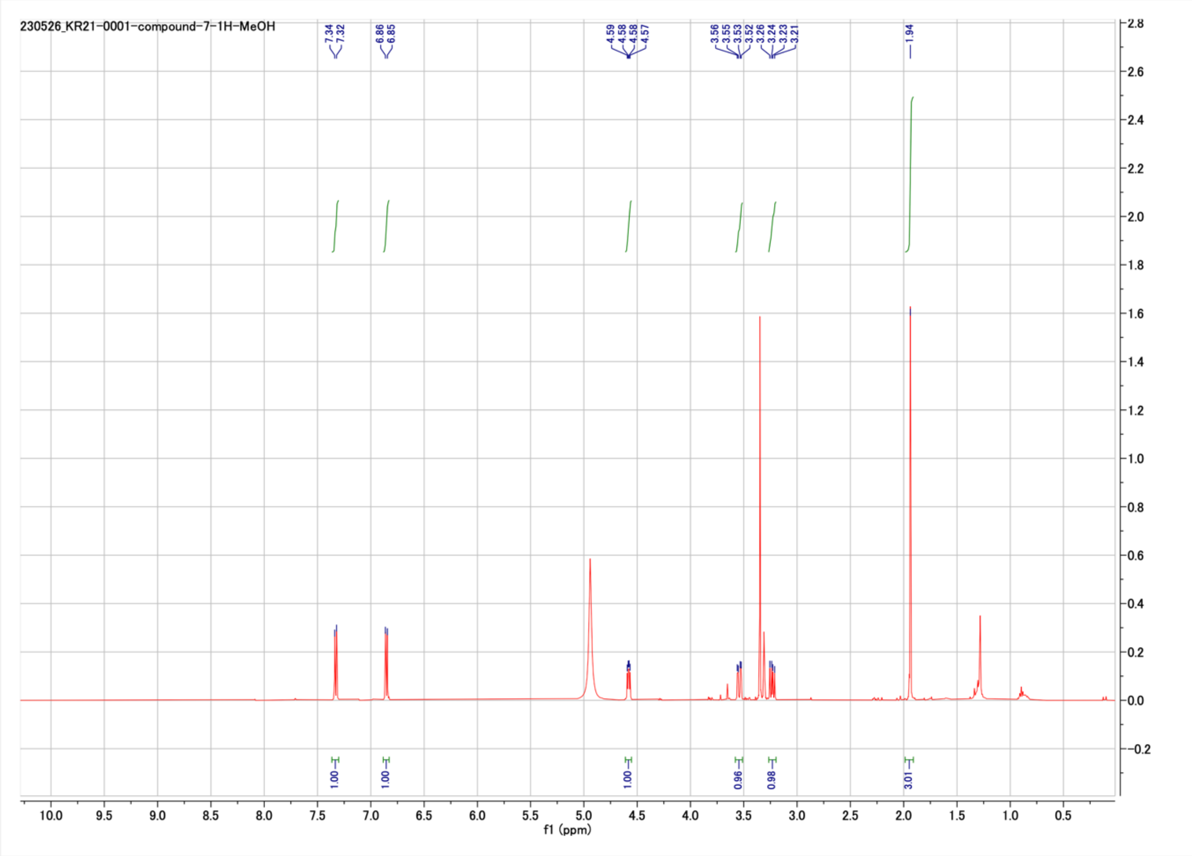

Figure S1: ^{1}H NMR spectrum of KR21-0001A (**1**) in CD_3OD.

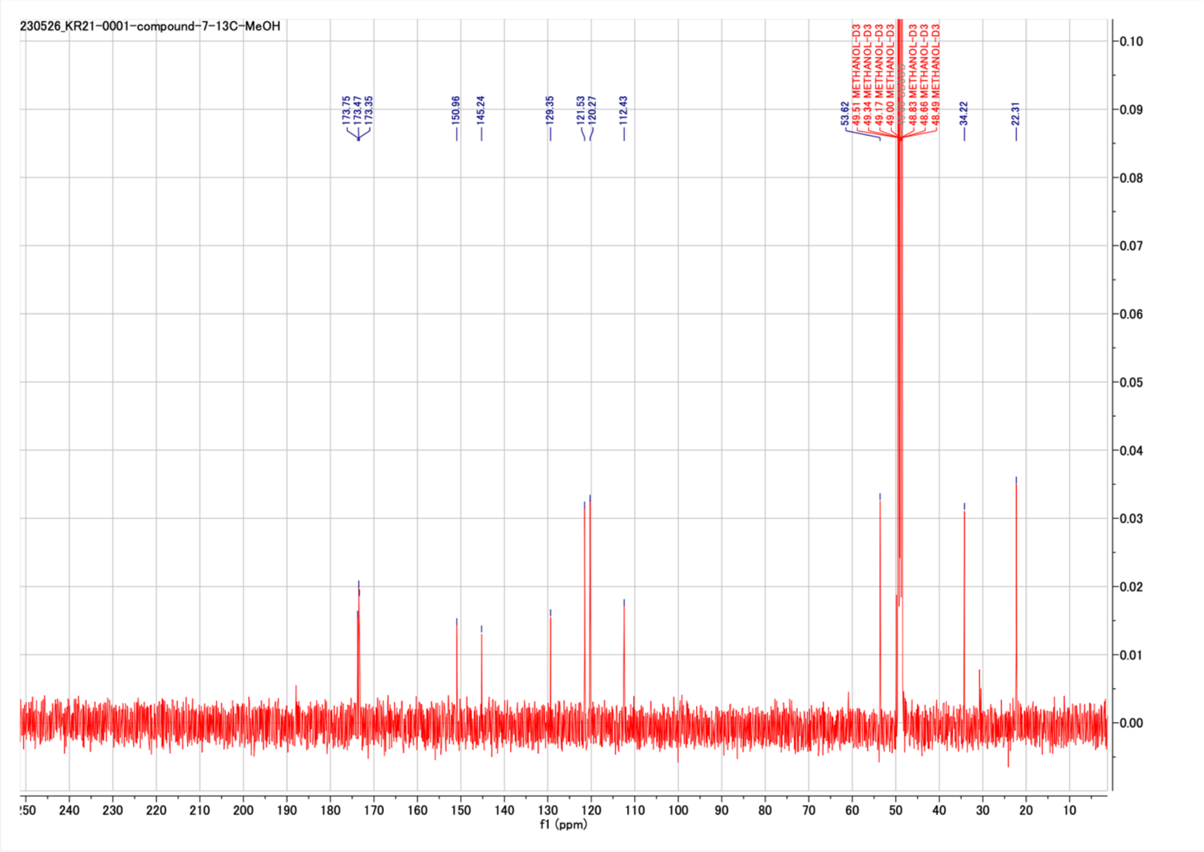

Figure S2: ^{13}C NMR spectrum of KR21-0001A (**1**) in CD_3OD.

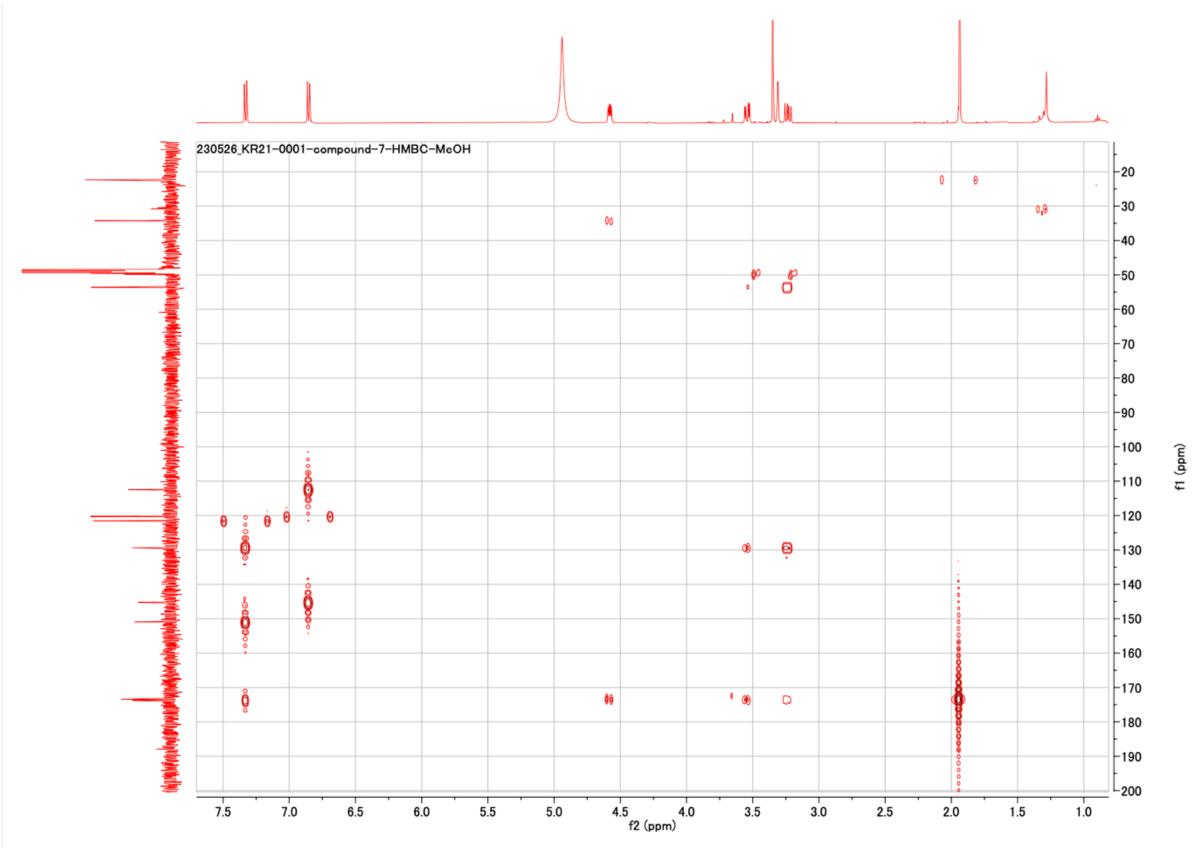

Figure S3: HMBC spectrum of KR21-0001A (**1**) in CD_3OD.

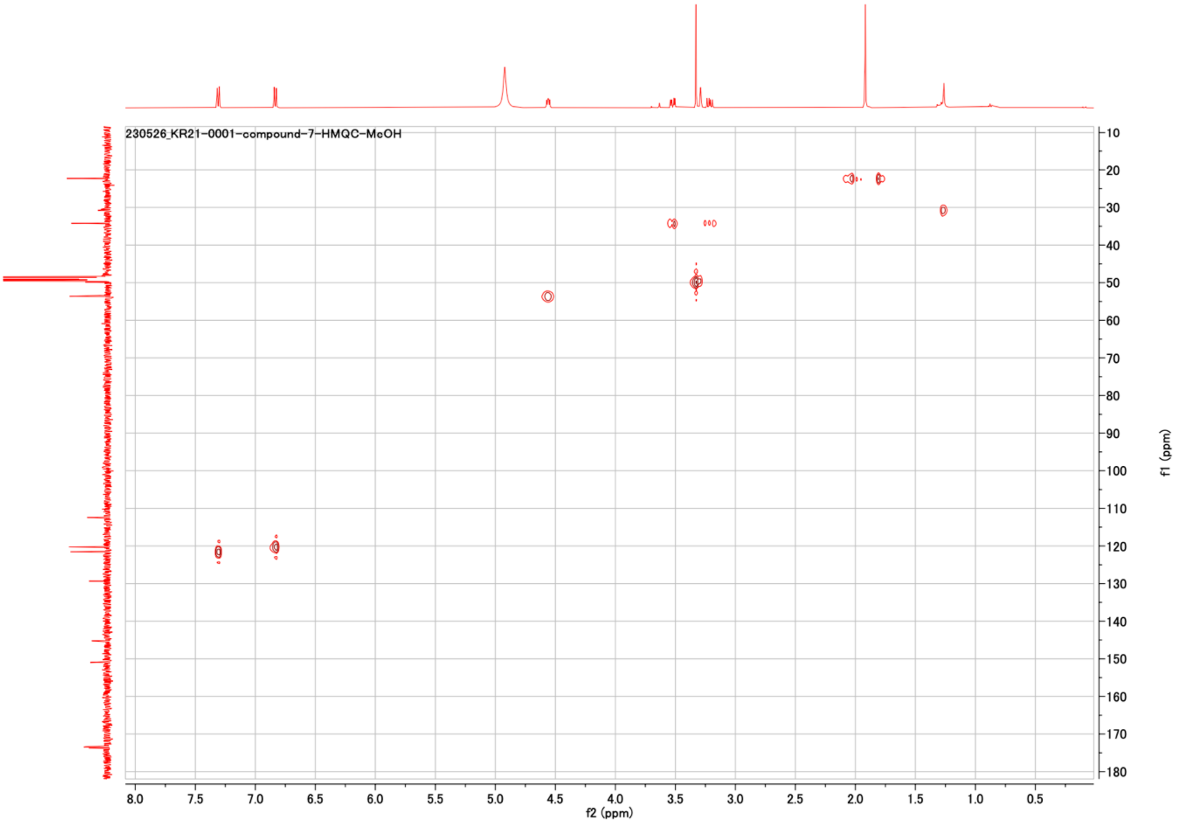

Figure S4: HMQC spectrum of KR21-0001A (**1**) in CD_3OD.

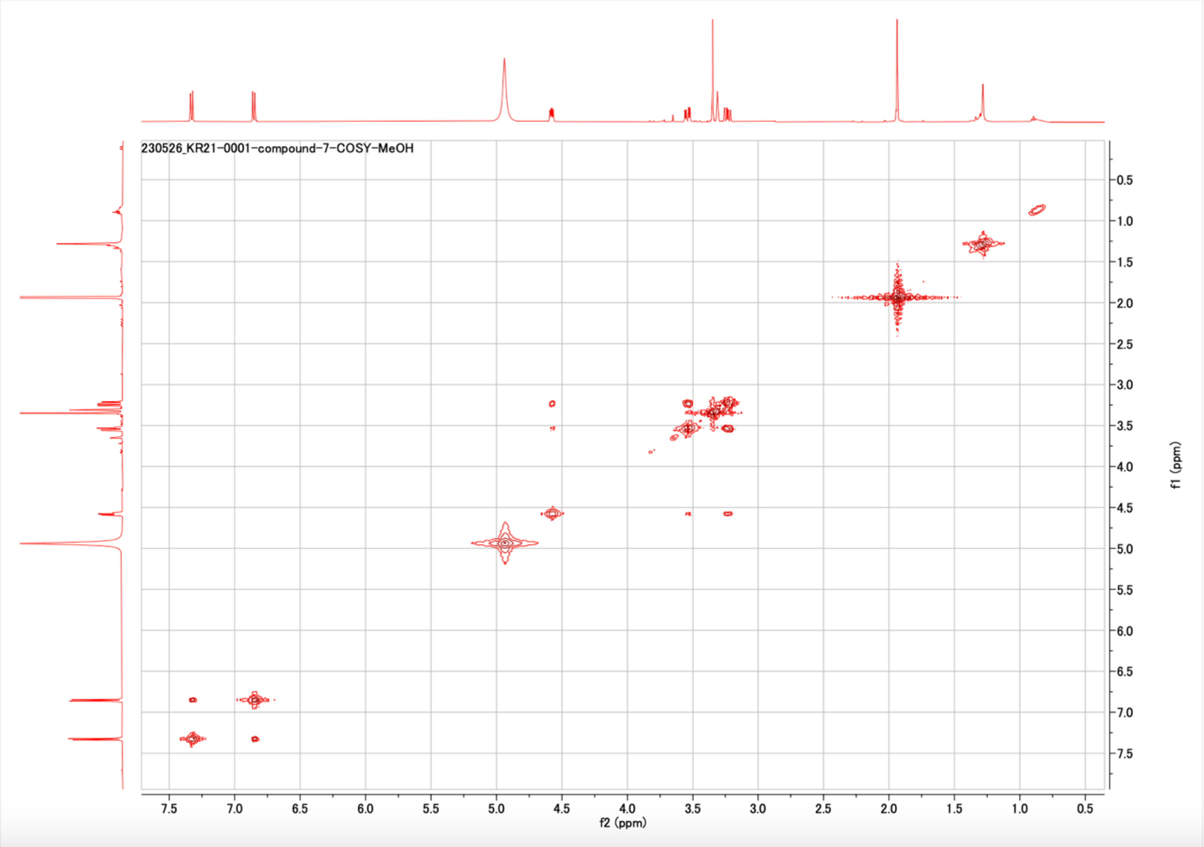

Figure S5: $^{1}H,^{1}H$ COSY spectrum of KR21-0001A (**1**) in CD_3OD.

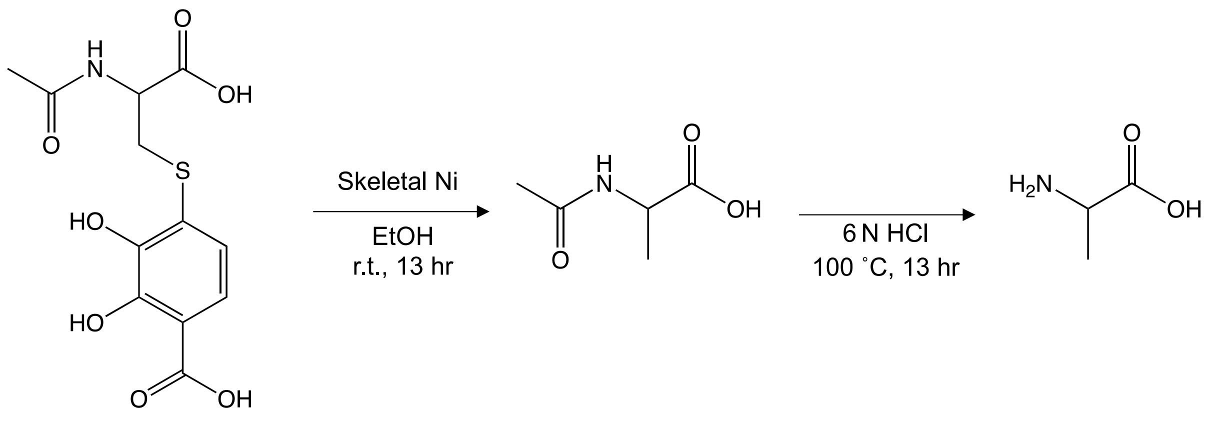

Figure S6: De-sulfurization and hydrolyzation of KR21-0001A (**1**).

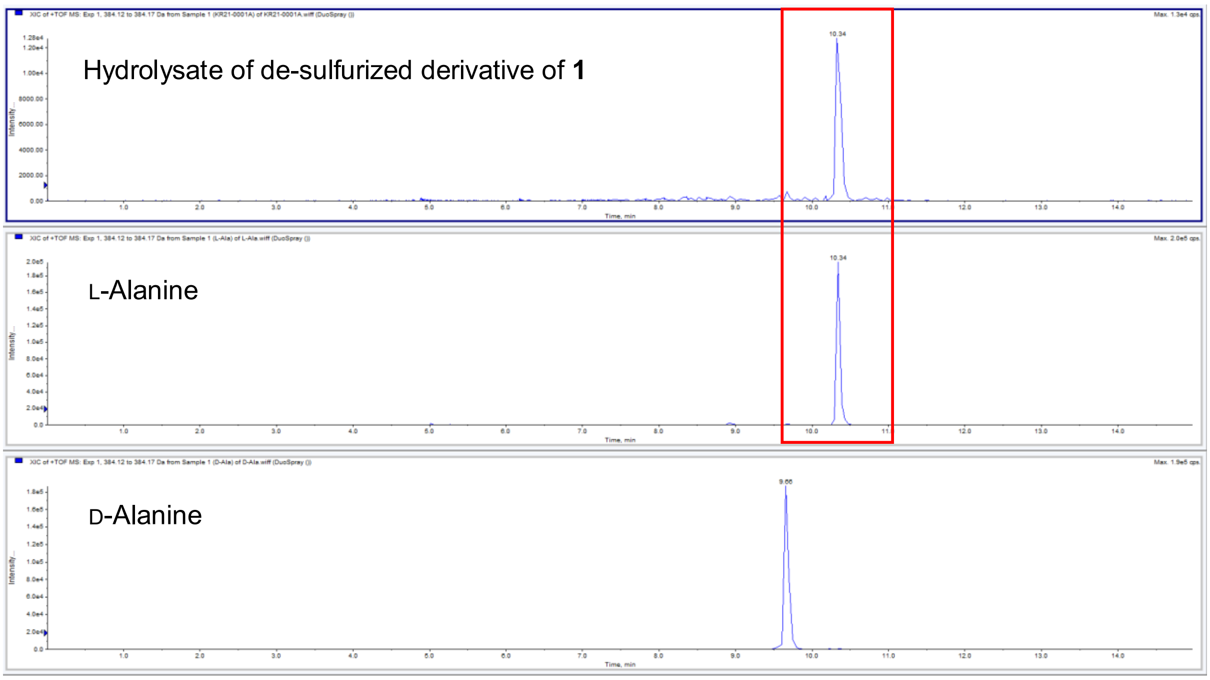

Figure S7: LC/MS analyses of the D-FDLA-derivatives.

Table S1: Physicochemical properties of KR21-0001A (**1**).

Properties	KR21-0001A
Appearance	Yellow oil
Molecular formula	$C_{12}H_{13}NO_7S$
Molecular weight	315
HR-ESI-MS (*m/z*)	
Calcd.	316.0485 $[M+H]^+$
Found	316.0484 $[M+H]^+$
UV_{max}^{MeOH} λ nm (ε)	215 (21389), 286 (10238)
IR ν cm^{-1}	3247, 2920, 2847, 2520, 1720, 1652, 1605, 1543, 1450, 1428, 1373, 1291, 1223, 1170, 1147, 1044, 1024, 982, 964, 905, 823, 750
$[\alpha]_D^{23}$ (c 0.1, MeOH)	-11.9
Soluble	MeOH, DMSO
